# Recurrence of brain arteriovenous malformations in pediatric patients: a long-term follow-up study

**DOI:** 10.1007/s00701-023-05612-8

**Published:** 2023-05-04

**Authors:** Elias Oulasvirta, Päivi Koroknay-Pál, Jussi Numminen, Ahmad Hafez, Rahul Raj, Behnam Rezai Jahromi, Mika Niemelä, Aki Laakso

**Affiliations:** 1 Department of Neurosurgery, Helsinki University Hospital and University of Helsinki, Helsinki, Finland; 2 Department of Radiology, Helsinki University Hospital and University of Helsinki, Helsinki, Finland

**Keywords:** AVM, Arteriovenous malformation, Recurrence, De-novo, Pediatric, Long-term follow-up

## Abstract

**Background:**

Previously thought to be congenital, AVMs have shown evidence of de-novo formation and continued growth, thus shifting thoughts on their pathophysiology. Pediatric AVM patients have been reported to be more prone to develop AVM recurrence after a seemingly complete cure. Therefore, we assessed the risk of AVM treated in childhood to recur in adulthood after a long-term follow-up in our own cohort.

**Methods:**

Control DS-angiography was arranged during 2021–2022 as part of a new protocol for all AVM patients who were under 21 years of age at the time of their treatment and in whom the treatment had occurred at least five years earlier. Angiography was offered only to patients under 50 years of age at the time of the new protocol. The complete eradication of AVM after the primary treatment had been originally confirmed with DSA in every patient.

**Results:**

A total of 42 patients participated in the late DSA control, and 41 of them were included in this analysis after excluding the patient diagnosed with HHT. The median age at the time of admission for AVM treatment was 14.6 (IQR 12–19, range 7–21 years) years. The median age at the time of the late follow-up DSA was 33.8 years (IQR 29.8–38.6, range 19.4–47.9 years). Two recurrent sporadic AVMs and one recurrent AVM in a patient with hereditary hemorrhagic telangiectasia (HHT) were detected. The recurrence rate was 4.9% for sporadic AVMs and 7.1% if HHT-AVM was included. All the recurrent AVMs had originally bled and been treated microsurgically. The patients with sporadic AVM recurrence had been smoking their whole adult lives.

**Conclusions:**

Pediatric and adolescent patients are prone to develop recurrent AVMs, even after complete AVM obliteration verified by angiography. Therefore, imaging follow-up is recommended.

## Introduction

The leading cause of hemorrhagic stroke in the pediatric population is a ruptured brain arteriovenous malformation (AVM) [6]. Furthermore, pediatric AVM patients present with rupture more often than adults [27]. In a recent large single-center study, 16% of all AVM patients were children [27]. Previously thought to be congenital, AVMs have shown evidence of de novo formation and continued growth, thus shifting thoughts on their pathophysiology. A recent landmark study showed that sporadic brain AVMs are associated with activating mutations on endothelial KRAS that lead to dysregulation of the MAPK–ERK pathway [26]. Further studies have identified other activating mutations downstream in the MAPK-ERK pathway from the AVM samples [3, 13, 15]. Also, smoking seems to play a role in the growth of AVMs [30], as has been shown in cerebral aneurysms [21].

Once complete eradication or occlusion of AVM has been verified by digital subtraction angiography (DSA), no further long-term angiographic follow-up has been generally warranted in adult patients. However, there is diversity in the guidance on following pediatric patients after successful AVM treatment. Some studies suggest that the first angiographic follow-up with magnetic resonance angiography (MRA) [22] or with DSA [25] should take place as soon as one year after the treatment, the main focus being on the diagnosis of the residual AVM missed in the initial postoperative angiography. Other published studies propose angiographic follow-up yearly until adult age with MRA [1] or with DSA at one, three, and five years and thereafter every five years [25]. In pediatric patients with DSA-verified complete eradication or occlusion, the percentage of patients harboring recurrent AVMs has varied between 6% and 28% [1, 4, 7, 20, 22, 24, 25]. The recurrence was most likely to occur in patients with diffuse-type AVMs [20] and in those with deep venous drainage [7, 24]. In a recent single-center study by Aboukais et al., all the recurrent AVMs in a patient cohort occurred among pediatric patients [1]. However, the follow-up time of all the above studies was somewhat limited, ranging from 14 months to 9 years [1, 7, 20, 22, 24, 25]. In a recent review study, the first recurrence was detected at three months and the last recurrence at 17 years post-treatment, the average time to recurrence being five years [18].

In this study, we aimed to assess the risk of late AVM recurrence in patients treated during childhood or early adulthood that displayed complete eradication or occlusion after the initial treatments. We conducted the long-term follow-up with DSA as the sensitivity of MRA may not always be sufficient in detecting small recurrent brain AVMs [17]. We hypothesized that late recurrence would be rare in these patients.

## Methods

### Patients

We retrospectively collected data on pediatric AVM patients admitted to the Department of Neurosurgery, Helsinki University Hospital (Helsinki, Finland), during 1985–2012. Traditionally, we have not conducted routine long-term follow-up angiographic imaging in asymptomatic patients with AVMs in which complete eradication was verified by DSA. On the basis of increasing clinical experience of recurrences, however, we introduced a follow-up protocol in January 2021 comprising a clinical visit and DSA study for patients treated in childhood or early adulthood (<21 years) to evaluate the long-term risk of AVM recurrence. The control DSA study was offered to patients under 50 years of age at the time of follow-up, as possible asymptomatic recurrences would probably not have led to any interventions in those older than 50 years.

We did not include extracranial AVMs; thus, the abbreviation “AVM” in this study refers only to brain AVMs. All AVM diagnoses were confirmed by digital subtraction angiography (DSA). AVM size, location, angioarchitecture, and associated aneurysms were evaluated from DSA and, if relevant, from computer tomography angiography (CTA) and magnetic resonance images (MRI). We scrutinized the patients’ medical records, including relevant radiological images (if existing in digital format, the digital Picture Archiving and Communication System was introduced in 1998–1999). All patients had a post-treatment DSA after the last treatments that confirmed complete AVM eradication or occlusion.

### Follow-up imaging

Patients fulfilling the new follow-up protocol criteria were contacted by letter and offered a clinical follow-up visit, followed by a DSA study if the patient gave consent after counseling. All patients that underwent DSA had a complete four-vessel DSA study (bilateral common carotid artery (CCA) and bilateral vertebral artery (VA) and, if deemed necessary, separate internal carotid artery (ICA) and external carotid artery (ECA) angiographies. An experienced neuroradiologist and neurosurgeon independently analyzed all DSA studies.

### Statistical analysis

Due to the relatively small number of patients, we did not conduct any statistical intergroup comparisons. We present categorical variables as absolute numbers with percentages. We analyzed continuous data for skewness, and since all data were non-parametrically distributed, we present continuous data as medians with interquartile ranges and minimum and maximum ranges. We performed the statistical analysis using SPSS software version 24.0 (IBM Inc, Armonk, New York). One patient with an HHT-related AVM is presented separately and excluded from the analyses.

## Results

### Patients and symptoms

**Fig. 1:**
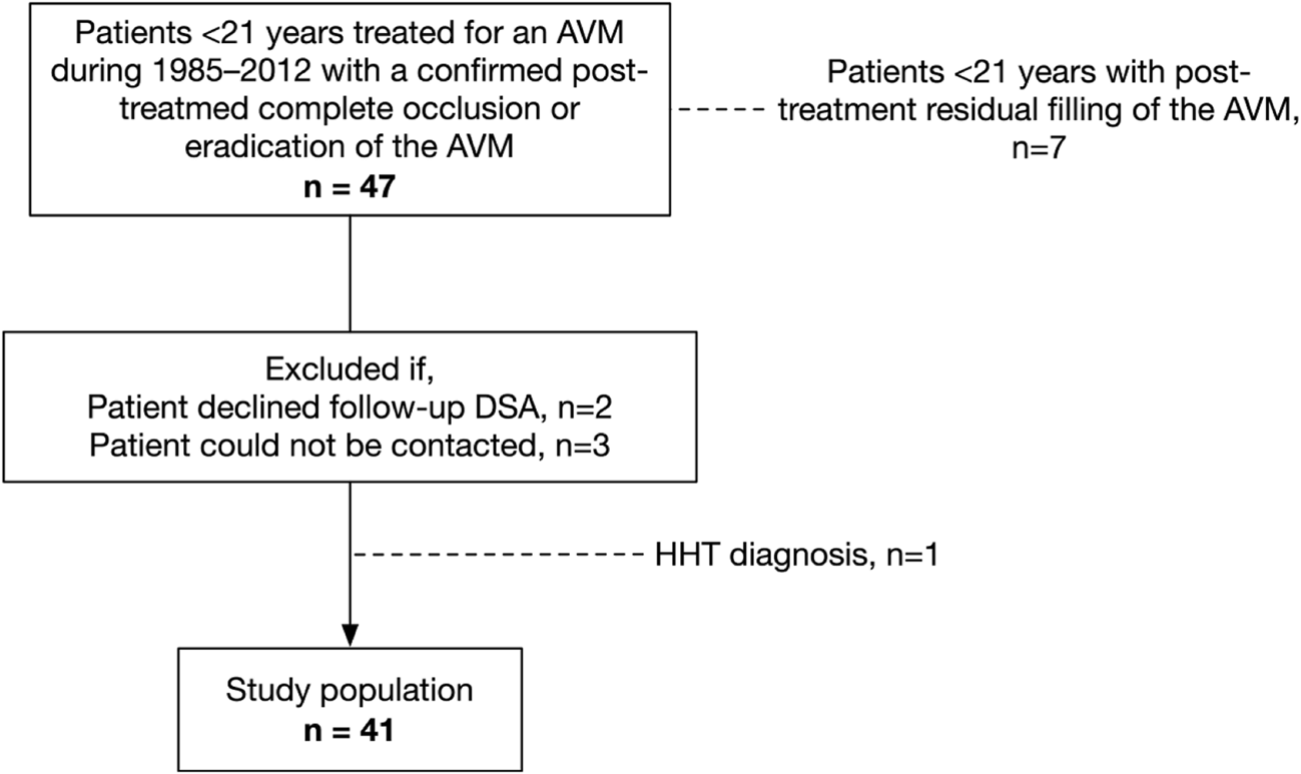
Flowchart of patient selection

Of 54 patients treated for AVM under the age of 21, 47 had a completely occluded or eradicated AVM. Of these 47 invited patients, three could not be reached, and two declined to participate in the DSA study. A total of 42 patients participated in the late DSA control, and 41 of them were included in this analysis after excluding the patient diagnosed with HHT (who is presented separately) (Fig. 1). The control DSA studies were performed between March 2021 and February 2022. The median age at the time of admission when starting AVM treatment was 14.6 years (IQR 12–19, range 7–21 years). The index admissions took place during 1985–2012. The median age at the time of the late follow-up DSA was 33.8 years (IQR 29.8–38.6, range 19.4–47.9 years). Two-thirds of the patients were male. In 71% of the patients, AVM rupture occurred before the obliteration, whereas 63% presented with a rupture. Epilepsy was a presenting symptom in 29% of patients (Table 1).

**Table 1:** Patient and AVM characteristics

	Recurrence	No recurrence	Total
	*N* = 2	*N* = 39	*N* = 41
*Patient characteristics*			
Median age at treatment, y	17.0	14.4	14.5
Median age at time of study	36.5	33.0	33.8
Male	2 (100%)	25 (64%)	27
Smoking	2 (100%)	8 (21%)	35*
Presentation			
Rupture	2 (100%)	27 (69%)	29
Epilepsy (not related to rupture)	1 (50%)	11 (28%)	12
*AVM characteristics*			
Exclusive deep venous drainage	1 (50%)	9 (23%)	10
Superficial venous drainage	1 (50%)	25 (64%)	26
Superficial + deep venous drainage	0 (0%)	5 (13%)	5
Left side	1 (50%)	15 (39%)	16
Superficial location	2 (100%)	32 (82%)	34
Supratentorial location	2 (100%)	35 (90%)	37
Spetzler-Martin grade			
1	0 (0%)	8 (21%)	8
2	1 (50%)	12 (32%)	13
3	1 (50%)	14 (37%)	15
4	0 (0%)	3 (8%)	3
5	0 (0%)	1 (3%)	1

*Data on six patients’ smoking status were missing

### Follow-up and AVM recurrence

The total follow-up time was 761 years. The median time from admission to follow-up DSA was 19.1 years (IQR 13.9–22.8, range 8.6–35.7 years). Three out of 42 patients demonstrated AVM recurrence at follow-up DSA, of which one was HHT-related AVM (4.9% recurrence rate for sporadic AVMs and 7.1% for the entire 42-patient cohort including the HHT-related AVM). Of the two patients with a recurrent AVM (excluding the HHT patient), the initial Spetzler-Martin grades were II and III, and both AVMs had bled before the obliteration. Both patients were male and active smokers. Both sporadic AVM recurrences in our study were also seen in MRI + 3D time-of-flight (TOF)-MRA performed after the DSAs. HHT-related small recurrent AVM was not observable in the MRI + 3D TOF-MRA imaging performed 15 months before the DSA.

### AVM characteristics

The median size of AVM nidus (in greatest dimension) was 29 mm (IQR 19.5–40.3, range 8–65 mm, Table 1). AVMs were classified as superficial in 34 (83%) patients and deep in seven (17%) patients. Deep venous drainage was present in 15 (37%) patients. AVM was located to the left in 16 patients (39%).

### Treatment

Most of the patients were surgically treated (90%), 14 patients (34%) received multimodality treatment, one patient (2%) was treated only with radiotherapy, and two patients (5%) were treated only with endovascular embolization. The treatment modalities are presented in greater detail and in comparison with recurrent AVMs in Table 2.

**Table 2:** Treatment modality

	Recurrence	No recurrence
	*N* = 2	*N* = 39
Microsurgery	1 (50%)	23 (59%)
Stereotactic radiotherapy	0 (0%)	1 (3%)
Embolization	0 (0%)	1 (3%)
Embolization + surgery	1 (50%)	11 (28%)
Embolization + surgery + radiotherapy	0 (0%)	2 (5%)
Embolization + radiotherapy	0 (0%)	1 (3%)

### Case illustrations of recurrent AVMs

1. A previously healthy 19-year-old man doing his military service experienced a sudden headache in the morning. The CT scan revealed an intracerebral hemorrhage (ICH) in the left temporal lobe. The patient was referred to the neurosurgical department. No neurological defects were observed, and the Glasgow Coma Scale (GCS) was 15. The DSA study demonstrated a somewhat diffuse left-sided temporal AVM with a maximum diameter of 2.5 cm. AVM received arterial feeding from the left posterior cerebral artery (PCA) and the left middle cerebral artery (MCA) and drained to the transverse sinus and straight sinus. The patient was operated on approximately one week after the presentation without any complications. DSA performed one day after the operation showed no signs of residual AVM. The patient recovered fully and, two months later, returned to finish his military service.

Almost 26 years later, the late follow-up DSA showed a recurrent AVM in the same location. The maximum diameter was 2.3 cm. It received arterial feeding from the left MCA, PCA, and external carotid artery (ECA) and drained to the vein of Galen and transverse sinus. MRI and 3D time-of-flight (TOF) MRA demonstrated the AVM, as well. The patient had been smoking from the age of 16 but stopped after the diagnosis of recurrent AVM. The patient had been completely asymptomatic for almost 26 years. At the time of the follow-up DSA, the patient was re-training for a new job and did not want to receive any additional treatment before finishing his studies. A follow-up has been scheduled.

2. In the second patient, a mild developmental delay accompanied by minor dysphasia and fine motor skills was observed at the beginning of childhood. Later in childhood, sensorineural hearing difficulty was diagnosed, and the patient received hearing aids for both ears. At the age of eight, he was operated on for a microform cleft lip.

**Fig. 2.**
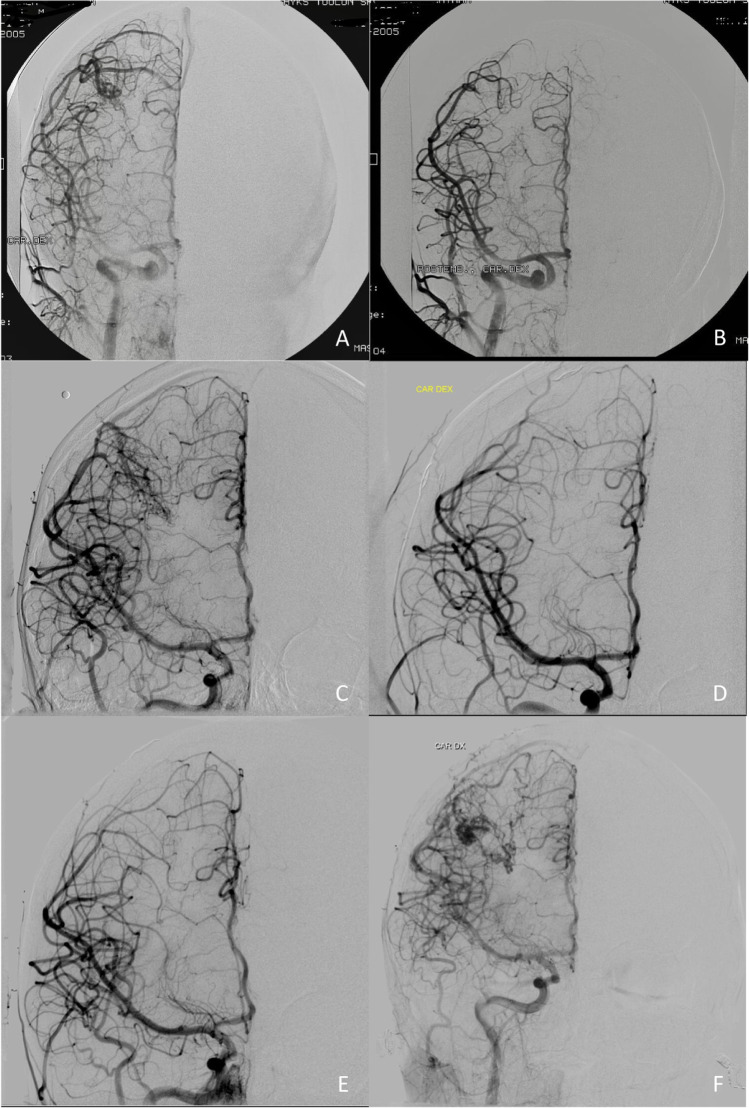
Digital subtraction angiographies (DSAs) of the second patient. The patient was referred to the Department of Neurosurgery at the age of 14, and surgery with pre-embolization was planned. (**A**) Pre-embolization DSA shows arteriovenous malformation (AVM) in the parietal Sylvian fissure on the right side. AVM received feeding from the middle cerebral artery (MCA) branches and drained via two large veins to the superior sagittal sinus (SSS). (**B**) After embolization, AVM did not fill up, and the early venous filling was no longer observed. (**C**) Six months later, the patient suffered a new generalized epileptic seizure. DSA showed recanalized AVM. (**D**) AVM was operated on a few months later. Postoperative DSA confirmed the complete eradication of AVM. (**E**) Surveillance DSA one year later showed no signs of AVM. The follow-up was concluded. (**F**) Fourteen years later, at the age of 30, the patient underwent DSA as part of a new follow-up protocol; it showed recurrent AVM in the same location. An elective operation was scheduled

The patient suffered his first generalized tonic-clonic seizure at the age of 11. Brain MRI and MRA were performed, which showed a right-sided parietal AVM in the Sylvian fissure. The patient was referred to the neurosurgical department at the age of 14. On admission, he did not show any signs of neurological problems. The DSA showed an AVM of approximately 2 cm in diameter that received arterial feeding from the MCA branches and drained via two large veins to the superior sagittal sinus (SSS). AVM was embolized during the same anesthesia, and it was planned to be operated on immediately. After the embolization, however, AVM filling and shunting were no longer observed, but one of the draining veins partly filled at the same time as the other veins. The operation was canceled, and control DSA was scheduled for 6–12 months later. Post-embolization brain CT showed no signs of bleeding, although minor bleeding cannot be ruled out because of the radio-opacity artifact from embolization. Six months later, before the follow-up DSA, the patient suffered a new generalized seizure. The brain MRA and the following DSA showed a recanalized AVM. No bleeding was observed. AVM was operated on a few months later, and during the operation, signs of old bleeding were observed. The operation was uneventful and postoperative DSA, done immediately, showed complete eradication of AVM. The one-year follow-up DSA performed showed no signs of AVM residual or recurrence. Fourteen years later, the late follow-up DSA showed recurrent AVM in the same location. An elective operation was scheduled. The patient had been smoking since the age of 14. He had been seizure free for many years and did not have any symptoms of the recurrent AVM (Fig. 2).

3. A previously healthy and normally developed 2-month-old boy was brought to the emergency department because of a sudden decrease in consciousness. He was intubated on the way to the hospital. The brain CT revealed a cerebellar intracerebral hemorrhage (ICH) and obstructive hydrocephalus. An external ventricular drain (EVD) was inserted. The DSA revealed a small AVM, 1 cm in diameter, that received arterial feeding from the posterior inferior cerebellar artery (PICA) and drained via one vein to the confluence of sinuses. The patient was operated on successfully, and immediate postoperative DSA showed no signs of residual AVM. The patient was left with minor ataxia, muscular hypotonia, lower limb weakness, and cognitive impairment. Now as an adult, the patient lives independently, walks without assistance, goes to the gym, and has completed a bachelor’s degree. At the age of 17, he was diagnosed with pulmonary AVM, which was incidentally found during a heart MRI. The genetic assessment revealed an ENG mutation, and the HHT diagnosis was confirmed. After the HHT diagnosis, brain MRI and TOF-MRA, performed in March 2020, showed no signs of recurrent AVMs. One year and three months later, the late follow-up DSA showed recurrent HHT-related AVM in the same location as the original AVM. The patient received stereotactic radiotherapy; follow-up continues.

## Discussion

Two recurrent sporadic AVMs and one recurrent HHT-AVM were detected in the long-term follow-up DSA of patients who were treated under the age of 21 for an unruptured or ruptured AVM. The recurrence rate in our study was 4.9% for sporadic AVMs and 7.1% if HHT-AVM was included; this is in line with the 4.8% reported in the latest meta-analysis of 1134 AVMs [23].

Recurrent sporadic AVMs were detected 26 years and 14 years after the previous angiographies. The patient who developed recurrence 14 years later had also had AVM recanalization six months after the primary treatment of endovascular embolization. After the recanalization, he was surgically treated, and the one-year control DSA showed no signs of AVM.

AVM rupture has been associated with later recurrence [9, 33], as was the case with the recurrent sporadic AVMs in our study. It has been postulated that AVM hemorrhage could cause localized vasoconstriction and lead to a hidden compartment that may be missed in the initial operation [29]. However, the AVM rupture in our second case was only observed in the elective operation performed later, contradicting the vasoconstriction hypothesis. It is also possible that the second patient’s AVM did not spontaneously rupture, and the bleeding observed in the surgical operation had occurred imperceptibly during embolization. Endothelial KRAS mutations have been associated in vitro with changes in vascular morphogenesis and disassembly of adherens junctions [12, 26], which could mean that ruptured AVMs are biomolecularly more active and therefore more prone to later recurrence. We hypothesized that a somatic mutation predisposing to AVM in these patients was probably still present in perinidal capillaries in the vicinity of the surgically removed AVM, but with angiographically normal appearance and without AV shunting, thus later giving rise to the observed recurrence.

The two patients who developed recurrent sporadic AVM had been smoking practically their whole lives (from their teenage years). While impossible to draw any conclusions only from these cases, it is still worth mentioning. We recently reported smoking to be more frequent in brain AVM patients than in the general population (48 vs. 19%) [30]. Nicotine is known to promote the secretion and expression of vascular endothelial growth factor (VEGF), which leads to activation of the same MAPK-ERK pathway that is involved in the pathogenesis of sporadic AVMs [2, 8, 10-12, 26].

### Timing

Despite the accumulating evidence of AVM recurrences in the pediatric population, there is no consensus on the optimal follow-up strategy for these patients. Lang et al. reported four recurrent AVMs at 50 weeks, 51 weeks, 56 weeks, and 60 weeks from initial resection, of whom two had had earlier negative angiograms three and six months postoperatively, but no later recurrences were detected at the five-year angiography [22]. However, Morgenstern et al. observed a recurrence six years after resection in a patient who had previously had a negative follow-up DSA at four years [25]. In our study, one patient initially had negative one-year postoperative DSA but developed recurrent AVM in the DSA control 17 years later. Before our study, recurrent AVMs had been reported up to 20 years after the initial AVM eradication in patients without routine follow-up imaging [19]. In our study, the longest recurrence was 25 years after the complete resection, but naturally, it could have been observable before had the follow-up DSA has been performed earlier.

### Imaging modality

One of the patients in the cohort study of Lang et al. had MRA performed 10 months after the AVM surgery because of a seizure, which showed no signs of AVM, but two months later, one year after the surgery, the DSA showed a Spetzler-Martin grade II AVM [22]. A similar finding was made in the Morgenstern study, where two of the patients with recurrent AVMs on angiography had previously had negative MRI/MRAs [25]. The sensitivity of gadolinium (Gd)-enhanced MRI and MRA for detecting AVMs has been reported to be 75% [17]. However, DSA is an invasive study, with a risk of femoral hematoma and pseudoaneurysm formation or even arterial dissection and stroke in rare instances [14]. Moreover, radiation exposure makes it suboptimal for young patients [28]. Even so, we did not observe any complications from the DSA studies we performed other than one non-life-threatening allergic reaction to iodine contrast. Both sporadic AVM recurrences in our study were also seen in MRI + 3D TOF-MRA imaging, but the HHT-related small recurrent AVM was not observable in the MRI + 3D TOF-MRA imaging performed 15 months earlier. While DSA and MRA imaging have their benefits and drawbacks, Huang et al. showed in a small study of 15 AVM patients a 100% sensitivity in identifying residual/recurrent AVMs using ferumoxytol-enhanced MRI (Fe-MRI) [16].

### Treatment modality

A recent meta-analysis suggested different AVM recurrence rates based on treatment modality, with the lowest rate of 0.7% in the radiosurgery group compared to 8.5% in microsurgery and up to 36.4% in endovascular embolization [23]. It was suggested that the low recurrence rate in the radiosurgery group could be due to patient selection, staged treatment, and delayed AVM obliteration, which could cause early recurrences to be reported as residual AVMs or radiosurgery’s ability to reduce neovascularization around AVM tissue. However, the high recurrence rate in the embolization group more likely represents recanalization than true recurrence.

### Theory of AVM recurrence pathophysiology

The perinidal capillaries of AVM have been reported to be abnormal, with the absence of blood-brain barrier components and markedly dilated lumens [5, 31, 36]. Yet, they are connected to the normal capillary network, arterioles, and venules in the surrounding brain parenchyma [31]. These perinidal capillaries most likely harbor the same mutations activating the MAPK-ERK pathway as resected AVMs. This could explain why deep venous drainage and diffuse nidus morphology have been associated with recurrent AVMs [20, 22], as perinidal capillaries may be left out more easily after the surgical excision of an AVM. The impact of smoking on the risk of AVM formation, rupture, and recurrences should be studied more closely, taking into account the effect of nicotine (and possibly many other substances in tobacco smoke) in the MAPK-ERK pathway [10]. Although it is not fully understood why children are more likely than adults to develop recurrent AVMs, possible reasons could be their immature vasculature and elevated circulating angiogenic growth factors [23, 32]. Immunochemistry studies have found increased levels of endothelial progenitor markers CD31, CD34, and CD105, as well as smooth muscle proliferative marker pERK in recurrent AVMs [34, 35].

Fish et al. demonstrated that KRAS gain-of-function mutations on endothelial cells result in the creation of brain AVMs in mouse and zebrafish models and that these lesions may be reversed by inhibiting MEK signaling [12]. In a 14-year-old girl with recurrent cerebral AVM, Walcott et al. detected a rare stop-gain mutation in SMAD9, which resulted in downstream abnormalities in the bone morphogenic protein (BMP) signaling pathway. The same mutation led to AVM formation in zebrafish models [37].

### Follow-up protocol

Based on our study and the current literature on recurrent AVMs, we have adopted the following pragmatic, general follow-up strategy in our center for AVM patients treated in childhood or early adulthood (<21 years).

1. Endovascularly treated patients should be followed with DSA at 6 months and 2 years and thereafter every 5 years until the last control is performed at the age of 21 or older.
2. Microsurgically treated patients should be followed with DSA every 5 years until the last control is performed at the age of 21 or older.
3. After radiosurgery, MRI+MRA are performed annually until the complete occlusion is suspected and then verified with DSA. Once complete occlusion is verified with DSA, patients should be followed with DSA every 5 years until the last control is performed at the age of 21 or older.
4. If AVM obliteration has been achieved less than 5 years before the age of 21, at least one 5-year control DSA and one 10-year control MRA are performed.
5. Our study does not provide enough evidence to suggest any specific follow-up protocols for patients with HHT-related AVMs. The risk of recurrence seems high, and the follow-up protocol should be tailored, preferably in multi-professional teams.

As the patients, circumstances, and lesions vary highly, individual planning on follow-up should always be considered first.

## Conclusions

Two recurrent sporadic AVMs and one recurrent HHT-AVM were detected in the long-term surveillance DSA of patients who were treated under the age of 21. The recurrence rate in our study was 4.9% for sporadic AVMs and 7.1% if HHT-AVM was included. All the recurrent AVMs had bled and were originally microsurgically treated. The two patients with sporadic AVM recurrence had been smoking their whole lives. While it is still largely unknown why young AVM patients are more susceptible than older patients to an increased risk of developing AVM recurrence, the phenomenon is real, and therefore, imaging follow-up is recommended.
